# Screening for Viral Nucleic Acids in the Cerebrospinal Fluid of Dogs With Central Nervous System Inflammation

**DOI:** 10.3389/fvets.2022.850510

**Published:** 2022-03-24

**Authors:** Renee M. Barber, Qiang Li, Jonathan M. Levine, Susan J. Ruone, Gwendolyn J. Levine, Patrick Kenny, Suxiang Tong, Scott J. Schatzberg

**Affiliations:** ^1^Department of Small Animal Medicine and Surgery, University of Georgia College of Veterinary Medicine, Athens, GA, United States; ^2^Becker Animal Hospital, Veterinary Centers of America, San Antonio, TX, United States; ^3^Department of Small Animal Clinical Sciences, Texas A&M University College of Veterinary Medicine and Biomedical Sciences, College Station, TX, United States; ^4^Division of HIV Prevention, Centers for Disease Control and Prevention, Atlanta, GA, United States; ^5^Department of Veterinary Pathobiology, Texas A&M University College of Veterinary Medicine and Biomedical Sciences, College Station, TX, United States; ^6^Department of Veterinary Clinical Sciences, The Royal Veterinary College, University of London, Hertfordshire, United Kingdom; ^7^Division of Viral Diseases, Centers for Disease Control and Prevention, Atlanta, GA, United States

**Keywords:** canine, central nervous system (CNS), inflammation, meningoencephalitis of unknown origin (MUO), virus, polymerase chain reaction (PCR), bunyavirus, La Crosse virus (LACV)

## Abstract

Central nervous system (CNS) inflammation is a common cause of neurological dysfunction in dogs. Most dogs with CNS inflammation are diagnosed with presumptive autoimmune disease. A smaller number are diagnosed with an infectious etiology. Additionally, at necropsy, a subset of dogs with CNS inflammation do not fit previously described patterns of autoimmune disease and an infectious cause is not readily identifiable. Because viral infection is a common cause of meningoencephalitis in people, we hypothesize that a subset of dogs presented with CNS inflammation have an occult viral infection either as a direct cause of CNS inflammation or a trigger for autoimmunity. The goal of this research was to screen cerebrospinal fluid from a large number dogs with CNS inflammation for occult viral infection. One hundred seventy-two dogs with neurological dysfunction and cerebrospinal fluid (CSF) pleocytosis were identified. Of these, 42 had meningoencephalitis of unknown origin, six had steroid-responsive meningitis-arteritis, one had eosinophilic meningoencephalitis, five had documented infection, 21 had and undetermined diagnosis, and 97 had a diagnosis not consistent with primary inflammatory disease of the CNS (e.g., neoplasia). CSF samples were subsequently screened with broadly reactive PCR for eight viral groups: adenovirus, bunyavirus, coronavirus, enterovirus, flavivirus, herpesvirus, paramyxovirus, and parechovirus. No viral nucleic acids were detected from 168 cases screened for eight viral groups, which does not support occult viral infection as a cause of CNS inflammation in dogs. La Crosse virus (LACV) nucleic acids were detected from four cases in Georgia. Subclinical infection was supported in two of these cases but LACV could not be ruled-out as a cause of infection in the other two cases, suggesting further research is warranted to determine if LACV is an occult cause of CNS inflammation in dogs.

## Introduction

Central nervous system (CNS) inflammation is a common cause of neurological dysfunction in dogs. Currently, at tertiary referral hospitals, the majority of dogs with CNS inflammation are diagnosed with presumptive autoimmune disease, or meningoencephalomyelitis of unknown origin (MUO) (1). The remainder are diagnosed with a variety of infectious etiologies, with bacterial, viral, protozoal, and fungal being the most common (1, 2). There is also a subset of cases that after histological evaluation of CNS tissue, do not fit previously described patterns of autoimmune disease and lack a readily identifiable infectious cause despite extensive searching with traditional histological stains, bacterial and viral culture, and PCR for previously described infectious organisms.

Several research groups have looked for occult infectious causes of CNS inflammation in dogs. Most notably, researchers have long hypothesized that MUO has an infectious cause, either directly leading to stimulation of an immune response or triggering autoimmunity (3). However, to date, an infectious etiology for MUO has not been identified (4–10). Large numbers of dogs with neurological dysfunction also have been screened for specific infectious agents such as *Anaplasma phagocytophilum, Borrelia burgdorferi sensu lato* (5), and *Bartonella* spp. (8) with negative results.

We hypothesize that a subset of dogs presenting for primary CNS inflammation have an occult viral infection. Viral infections are a common cause of meningoencephalitis in people (11), and there is strong support for virus-triggered CNS autoimmunity derived from animal models (12). Additionally, over the past several decades, there have been sporadic reports of uncommon viral infections leading to CNS disease in dogs (13–20).

Diagnosis of viral meningoencephalitis in people is often accomplished by PCR of cerebrospinal fluid (CSF) (11), and broadly reactive PCR methodologies have repeatedly proven useful in viral discovery (21, 22). Based on this, we utilized broadly reactive PCR assays to interrogate cerebrospinal fluid (CSF) from dogs with evidence of CNS inflammation for detection of pathogens in 8 viral groups (adenovirus, bunyavirus, coronavirus, enterovirus, flavivirus, herpesvirus, paramyxovirus, and parechovirus), all of which have been documented as causative agents of meningoencephalitis in people (23–30).

## Materials and Methods

### Case Samples

CSF was collected in accordance with Institutional Animal Care and Use guidelines in routine fashion from the cerebellomedullary or lumbar cistern from dogs that presented with neurological signs to the University of Georgia College of Veterinary Medicine (UGA-CVM), Texas A&M University College of Veterinary Medicine and Biomedical Sciences (TAMU-CVM), and The Royal Veterinary College (RVC), University of London between 2003 and 2008. Cytologic analysis and protein quantification were performed by a board-certified clinical pathologist. A separate aliquot of excess CSF, that was not subjected to clinical pathological testing and therefore still contained cells, was stored at −80°C. All samples were analyzed from 2008 to 2010.

Cases were included in the study if the dogs had neck or back pain and/or neurological deficits referrable to the CNS with concurrent CSF pleocytosis (defined as >5 white blood cells (WBC)/μl and <4,000 red blood cells/μl) (31). Clinical information, including age, gender status, breed, clinical history, neurological signs, neuroanatomic localization, magnetic resonance imaging (MRI) or computed tomography (CT) findings, clinical pathology results, presumptive diagnosis, treatment, response to treatment, and necropsy findings were recorded from medical records. Based on diagnostic results, dogs were categorized into one of three groups: (1) a diagnosis of primary inflammatory disease of the CNS, (2) a diagnosis not consistent with primary inflammatory disease of the CNS (e.g., neoplasia), and (3) a lack of a definitive diagnosis after MRI and CSF analysis and/or necropsy (Supplementary Table 1). Where possible, a necropsy diagnosis was utilized. When not possible, previously described clinical diagnostic criteria were utilized to diagnose non-infectious inflammatory disease (32–37), and infection was diagnosed based on culture, serology, PCR, and/or CSF cytology.

### Nucleic Acid Extraction and PCR Quality Control

Total nucleic acids were extracted from CSF (Qiagen MinElute Virus Spin Kit, Qiagen) and stored as single-use aliquots at −80°C. A 215 base pair (bp) fragment of the canine histone 3.3 gene (38) or 191 bp fragment of the glyceraldehyde 3-phosphate dehydrogenase (GAPDH) gene (39) was amplified from all samples to confirm DNA integrity. RNA integrity was confirmed in all samples by reverse transcription PCR (RT-PCR) amplification of superoxide dismutase (SOD) (expected product size 440 bp) (40). To avoid contamination, nucleic acid extraction, PCR preparation (pre-amplification), PCR, and sequencing were carried out in different rooms. Negative controls containing no DNA or RNA template were run in parallel with all PCR reactions. Additionally, mock nucleic acid extraction of sterile water was performed in parallel with all clinical cases and utilized as a negative control for all PCR reactions.

### Broadly Reactive Pan-Viral Group PCR

Consensus, degenerate, or consensus-degenerate hybrid primers were used for broadly reactive viral PCR (Table 1). Adenovirus PCR (Platinum *Taq* DNA Polymerase kit, Invitrogen); bunyavirus RT-PCR and coronavirus, flavivirus, and paramyxovirus semi-nested RT-PCR (SuperScript III One-Step RT-PCR System, Invitrogen); herpesvirus semi-nested PCR (snPCR) (HotStar*Taq* DNA Polymerase kit, Qiagen); and parechovirus and enterovirus real-time RT-PCR (rRT-PCR) (SuperScript III One-Step Quantitative RT-PCR System, Invitrogen) were performed according to manufacturer's instructions with a final volume of 50 μl and final primer concentration of 1 μM unless otherwise noted. RT-PCR reactions contained 20 U RNase inhibitor (Roche Diagnostics) and PCR and snPCR reactions used 200 μM (each) of deoxynucleotide triphospates (dNPTs) (Roche Diagnostics). Initial reactions were performed with 5 μl template DNA or RNA, and semi-nested reactions were performed with 2 μl of template from the initial reaction.

**Table 1:** Sequences of viral oligonucleotide primers use for polymerase chain reaction.

	**Primer sequence (5^**′**^ to 3^**′**^)**	**Amplicon** **(bp)**
Adenovirus ADVE2B F	TCMAAYGCHYTVTAYGGBTCDTTTGC	450
Adenovirus ADVE2B R	CCAYTCHSWSAYRAADGCBCKVGTCCA	
Adenovirus ADVhexon F	AARGAYTGGTTYYTGRTNCARATG	400
Adenovirus ADVhexon R	CCVAGRTCNGTBARDGYSCCCAT	
Bunyavirus BCS82C	ATGACTGAGTTGGAGTTTCATGATGTCGC	251
Bunyavirus BCS332V	TGTTCCTGTTGCCAGGAAAAT	
Coronavirus F2	ATGGGITGGGAYTATCCWAARTGTG	440
Coronavirus R3A	AATTATARCAIACAACISYRTCRTCA	
Coronavirus R3B	TATTATARCAIACIACRCCATCRTC	
Coronavirus R2A8	CTAGTICCACCIGGYTTWANRTA	199
Coronavirus R2B8	CTGGTICCACCI GGYTTNACRTA	
Flavivirus cFD2	GTGTCCCAGCCGGCGGTGTCATCAGC	250
Flavivirus MAMD	AACATGATGGGRAARAGRGARAA	
Flavivirus FS778	AARGGHAGYMCDGCHATHTGGT	214
Herpesvirus DFASA	GTTCGACTTYGCNAGYYTNTAYCC	500
Herpesvirus GDTD1B	GCATGCGACAAACACGGAGTCNGTRTCNCCRTA	
Herpesvirus VYGA	GTGCAACGCGGTGTAYGGNKTNACNGG	236
Paramyxovirus PAR-F1	GAAGGITATTGTCAIAARNTNTGGAC	650
Paramyxovirus PAR-F2	GTTGCTTCAATGGTTCARGGNGAYAA	
Paramyxovirus PAR-R	GCTGAAGTTACIGGITCICCDATRTTNC	563
Enterovirus AN350	GGCCCTGAATGCGGCTAATCC	145
Enterovirus AN 351	GCGATTGTCACCATWAGCAGYCA	
Enterovirus probe AN234	FAM-CCGACTACTTTGGGWGTCCGTGT-BHQ-1	
Parechovirus AN345	GTAACASWWGCCTCTGGGSCCAAAAG	194
Parechovirus AN344	GGCCCCWGRTCAGATCCAYAGT	
Parechvirus probe AN257	YY-CCTRYGGGTACCTYCWGGGCATCCTTC-BHQ-1	

Generic adenovirus primers previously designed (41) to an ~450 bp region of the DNA polymerase gene (AdVE2B F and AdVE2B R) and 400 bp region of the hexon gene (AdVhexon F and AdVhexon R) were used for PCR as previously described (4). Canine adenovirus (CAV)-1 and CAV-2 DNA extracted from purified virus-infected tissue culture supernatant was used as a positive control.

Generic bunyavirus primers BCS82C and BCS332V previously designed to an ~251 bp region of the small segment were used for RT-PCR (42). After initial reactions at 60°C for 1 min, 45°C for 30 min, and 94°C for 2 min, RT-PCR cycled 40 times at 94°C for 15 s, 50°C for 30 s, and 72°C for 30 s, followed by a final elongation step at 72°C for 7 min. RNA from a mutated clone of La Crosse virus (LACV) was used as a positive control.

Pan-coronavirus primers previously designed to an ~440 bp region of the highly conserved polymerase 1b open reading frame were used for snRT-PCR (21). Primers F2, R3A (0.5 μM), and R3B (0.5 μM) were used for the initial reaction and F2, R2A8, and R2B8 were used for the semi-nested reaction. Reverse transcription began at 60°C for 1 min, 45°C for 30 min, and 94°C for 2 min, followed by 40 cycles at 94°C for 15 s, 50°C for 30 s, and 72°C for 30 s with a final elongation step at 72°C for 7 min. RNA from human coronavirus OC43 was used as a positive control (expected product size 199 bp).

Pan-flavivirus primers previously designed to an ~250 bp conserved region of the non-structural protein NS5 gene were utilized for snRT-PCR (25). Primers cFD2 and MAMD were used for the initial reaction and cFD2 and FS778 were used for the semi-nested reaction. Reverse transcription began at 60°C for 1 min, 42°C for 30 min, and 94°C for 2 min, followed by 40 cycles at 94°C for 30 s, 50°C for 30 s, and 72°C for 1 min with a final elongation step at 72°C for 7 min. RNA from a mutated clone of St. Louis encephalitis (SLE) virus was used as a positive control (expected product size 214 bp).

Pan-herpesvirus primers previously designed to an ~500 bp region of the DNA polymerase gene were used for snPCR (43). Primers DFASA and GDTD1B were used for the initial reaction and VYGA and GDTD1B were used for the semi-nested reaction. Both reactions began with an initial hot-start at 94°C for 2.5 min, followed by 50 cycles at 94°C for 1 min, 50°C for 1 min, and 72°C for 1 min, with a final elongation step at 72°C for 10 min. DNA from canine herpesvirus type 1 was used as a positive control (expected product size 236 bp).

Pan-paramyxovirus primers PAR-F1, PAR-F2, and PAR-R previously designed to an ~650 bp region of the polymerase L gene were used for snRT-PCR as previously described (44). Template RNA from human parainfluenza virus 2 was used as a positive control (expected product size 563 bp).

Previously designed enterovirus and parechovirus primers (0.4 μM each) and probes (0.2 μM each) (TaqMan, Applied Biosystems) previously designed based on highly conserved 5′ non-translated regions were used for rRT-PCR (45, 46). After initial reactions at 50°C for 30 min and 95°C for 10 min, rRT-PCR cycled 50 times with the following parameters: 95°C for 15 s, 58°C for 30 s, and 72°C for 10 s, with probe detection during the 58°C annealing step (Roche LightCycler, Roche Diagnostics). Threshold cycle values were determined using commercially available software (Roche LightCycler, Roche Diagnostics). Template DNA from echovirus 30 and human parechovirus 1 (Harris strain) were used as positive controls for enterovirus and parechovirus rRT-PCR, respectively.

### Sequencing

PCR products were analyzed by 2% agarose gel electrophoresis with ethidium bromide staining under ultraviolet exposure, and amplicons were purified (MinElute PCR Purification Kit and QIAquick Gel Extraction Kit, Qiagen) for sequencing. Purified amplicons or plasmids were sequenced (BigDye Terminators v3.1 and ABI 3730xl, Applied Biosystems) using the corresponding amplification primers. Viral species were defined by comparison of DNA sequences with GenBank database entries using the Basic Local Alignment Search Tool (BLAST 2.0).

## Results

Cerebrospinal fluid from 172 pure or mixed breed dogs was tested by all PCR methodologies, including 92 from UGA-CVM, 26 from TAMU-CVM, and 54 from RVC. Primary CNS disease was diagnosed in 54 dogs: 42 with MUO, 6 with steroid-responsive meningitis-arteritis (SRMA), 1 with idiopathic eosinophilic meningoencephalitis, and 5 with infection (two with Rocky Mountain spotted fever, one with *Bartonella vinsonii*, one with Zygomycetes encephalitis, and one with encephalitis secondary to bacterial abscesses). Non-inflammatory CNS disease was diagnosed in 97 dogs. A diagnosis was not reached in 21 dogs.

All positive controls produced the expected PCR results; all negative controls were free of viral amplicons. Histone or GAPDH and SOD were amplified successfully from all cases. Nucleic acids from pan-viral group PCR for adenoviruses, coronaviruses, enteroviruses, flaviviruses, herpesviruses, paramyxoviruses, and parechoviruses were not detected in the 172 samples evaluated by PCR. Amplicons of the expected size (251 bp) (42) were detected by generic pan-bunyavirus RT-PCR in one case with MUO, two dogs with non-inflammatory CNS disease, and one dog with an open diagnosis. Direct sequencing of the amplicons from all cases demonstrated >95% sequence identity to the nucleoprotein gene of several LACV strains, including 65/OH-M (GenBank accession GU206123.1), 97/NC-M (GenBank accession GU206126.1), 93/MO-H (GenBank accession GU206138.1), 74/NY-M (GenBank accession GU206141.1), 00/WV-M (GenBank accession GU206147.1), and 00/NC-M (GenBank accession GU206111.1).

All LACV-positive cases were evaluated by the neurology service at the UGA-CVM. The positive MUO case was a 3-year-old male intact Boston terrier dog that was presented for evaluation of acute-onset blindness and an abnormal gait. The general physical exam, serum biochemistry, complete blood count, and thoracic radiographs were normal aside from a mild leukocytosis characterized by a mature neutrophilia. Neurological exam was consistent with multifocal CNS disease. No abnormalities were identified on magnetic resonance imaging (MRI) of the brain and cervical spinal cord. CSF from the cerebellomedullary cistern revealed 69 RBC/μl, 20 WBC/μl, characterized by a lymphocytic pleocytosis, and a total protein concentration of 16.5 mg/dl. There was complete resolution of clinical signs after treatment with prednisone and cytosine arabinoside. The other cases included an 11-year-old Boxer dog with seizures secondary to an insulinoma, a 12-year-old Weimaraner with seizures secondary to a nasal adenocarcinoma invading the olfactory bulb and frontal lobe of the brain, and an 11-year-old Shih Tzu with epilepsy and a normal MRI.

## Discussion

We tested a large number of dogs with CSF pleocytosis for occult viral infections using broadly reactive PCR. No viral nucleic acids were detected in CSF for seven of the viral groups evaluated: adenovirus, coronavirus, enterovirus, flavivirus, herpesvirus, paramyxovirus, parechovirus, which does not support occult viral infection as a cause of CNS inflammation in dogs. A small number of cases from the University of Georgia were positive for LACV. Subclinical infection was supported in two of these cases but LACV could not be ruled-out as a cause of infection in the other two cases, suggesting further research is warranted to determine if LACV is an occult cause of CNS inflammation in dogs in endemic regions of the United States.

The findings shared here are consistent with previous research reports. In recent research studies, no occult infectious organisms have been identified in dogs with MUO (7, 9, 10) or CNS inflammation (5, 8). However, this report expands on published research in several ways. First, CSF from a larger number of cases than has been previously reported was evaluated using broadly reactive molecular methodologies (i.e., interrogation for a large number of viral groups by broadly reactive PCR intended to identify all species within each group). Also, we included cases with CSF pleocytosis that did not have a readily identifiable diagnosis (and as such may have been more likely to have an occult viral infection).

The predominately negative results presented here could be due to study limitations. It is possible that screening a larger number of cases from a more geographically diverse population would yield positive results. There also were only a small number of dogs <1 year of age included in the final analysis. This was likely a reflection of the referral populations included in the study but may have precluded identification of viruses that would be expected to affect younger patients (13, 47). Additionally, a large number of cases screened (*n* = 97) had a diagnosis not consistent with primary CNS inflammation. These cases were included as a control population that might have blood-CSF barrier breakdown and thus be susceptible to contamination from bloodborne agents.

Panviral group (family and genera) PCR was chosen for this study due to its sensitivity in viral discovery (21, 22). Although next generation sequencing is ideal to identify a broad range of microorganisms, including those not yet known, the depth of sequencing may limit sensitivity and necessitate enrichment of viral nucleic acids to improve outcomes (48–52). However, despite the sensitivity of PCR, false negative results are still possible in this study for a number of reasons. For example, it is possible that viruses targeting CNS parenchyma could be missed in CSF. Using CSF with an elevated cell count and total protein has been shown to be important in humans when searching for viruses. For herpes simplex virus, screening of CSF samples requires a minimum of 5 cells/μl and >50 mg/dl protein to increase yield (53). Although all cases in our study had pleocytosis, we did not use elevated protein as an inclusion criterion.

Future studies could include larger numbers of dogs with CNS inflammation and assessment of brain parenchyma concurrent with CSF using a combination of methodologies such as next generation sequencing and panviral PCR to improve sensitivity. Clinicians and pathologists should work together to save fresh frozen brain tissue from all potential inflammatory cases. This would allow researchers to thorough molecular interrogation of larger numbers of dogs with MUO (9) and allow additional evaluation of the subset of dogs that have CNS inflammation but no definitive diagnosis upon completion of necropsy.

The LACV-positive findings in this report could represent false positive PCR results, evidence of subclinical infection, or suggest that LACV is a more common cause of CNS inflammation in dogs than previously recognized. LACV is an arbovirus known to cause disease in a variety of mammals in the Midwestern, mid-Atlantic, and southeastern United States (54). Although the majority of infections in people are suspected to be asymptomatic or result in mild, flu-like illness (54–59), true incidence is hard to quantify because diagnostic testing for LACV is not performed in these cases. In the United States, 50–150 cases of more severe LACV infection are reported each year (Centers for Disease Control and Prevention)^1^, with >90% representing neuroinvasive disease in children under age 16 (54, 60–62). Neuroinvasive disease has also been reported in numerous species (54), with necrotizing meningoencephalitis secondary to LACV infection documented in in five puppies (13) and one adult dog (63), all from Georgia.

False positive PCR results are considered unlikely in this study for several reasons. First, sequencing of PCR products confirmed specific amplification of LACV. Second, all PCR was conducted in a PCR-dedicated laboratory where PCR preparation and sample handling were physically separated from analysis of PCR products. Additionally, all PCRs were run in a laboratory that had not previously amplified or worked with LACV, and each PCR was run with a negative control to ensure no contamination was present (64, 65).

Subclinical infection is considered likely in the two cases that had neoplasia (invasive nasal adenocarcinoma and insulinoma) as a diagnosis for the cause of seizures and also may be possible the other two positive cases (one with MUO and one with seizures but a normal MRI). As dicussed, false positives are considered unlikely and asymptomatic infection has been documented in people. Although seroprevalence studies do not exist for dogs or people in Georgia, seroprevalence in people has been documented in neighboring states. It has been reported as 9.7% (66) and 11.3% (56) in the general population of North Carolina and 0.5% in the general population of Tennessee (67). Additionally, 22.7% of park employees tested in North Carolina and Tennessee were seropositive (68, 69).

Unfortunately, given the nature of this study, it is not possible to determine if LACV was a causative agent of disease in the dog diagnosed with MUO or the dog with late onset epilepsy of unknown cause. The dog with MUO improved with immunosuppressive therapy and the dog with epilepsy responded to treatment with anti-seizure medications. In the previous reports of canine LACV meningoencephalitis, all animals were severely affected, resulting in spontaneous death with pathology that demonstrated a severe, necrotizing meningoencephalitis (13, 63). However, five of these reported cases were puppies <2 weeks of age that lacked developed immune systems (13), and the adult dog described did initially respond to treatment with corticosteroids before relapsing 2 weeks later (63). Additionally, a spectrum of neuroinvasive disease has been documented in children. Reported signs on hospital admission include headache, vomiting, disorientation, seizures, but not all cases require hospitalization and the majority of cases have normal cross sectional imaging (70).

Further research into LACV as a possible cause of CNS inflammation in dogs should be considered. This research could be 2-fold. First, to document LACV seropositivity in the general population of dogs in endemic states. Second, to prospectively test dogs with CNS inflammation for LACV, allowing for long-term monitoring of these patients as well as additional LACV-specific diagnostic tests where indicated. This research will be hampered by the fact that there are no commercially available serological tests for LACV in dogs, leaving PCR and virus isolation as possibilities for clinical cases. A previously developed neutralization assay may aid in further serological evaluation of dogs for LACV antibodies (71).

Ultimately, this study limited evidence for previously unrecognized viral infections as a cause of CNS inflammation in dogs. However, the finding of LACV positive cases warrants further research in endemic areas. Moreover, considering that new, more sensitive technologies are constantly emerging and proving useful in viral discovery, researchers should continue to collect and preserve tissues from these cases for future analysis.

## Data Availability Statement

The raw data supporting the conclusions of this article will be made available by the authors, without undue reservation.

## Ethics Statement

The animal study was reviewed and approved by University of Georgia Clinical Research Committee.

## Author Contributions

SS contributed to conception and design of the study. ST contributed PCR methodology. RB, JL, GL, PK, and SS obtained clinical samples. RB, QL, and SR performed PCR studies. RB, ST, and SS supervised performance and analysis of PCR studies. RB wrote the first draft of the manuscript. All authors contributed to manuscript revision and approved the submitted version.

## Funding

This study was funded by grants from the Morris Animal Foundation (D07CA-152) and American Kennel Club Canine Health Foundation (1099).

## Author Disclaimer

The findings and conclusions in this report are those of the authors and do not necessarily represent the official position of the Centers for Disease Control and Prevention/the Agency for Toxic Substances and Disease Registry.

## Conflict of Interest

The authors declare that the research was conducted in the absence of any commercial or financial relationships that could be construed as a potential conflict of interest. The handling Editor declared a past co-authorship with one of the author RB.

## Publisher's Note

All claims expressed in this article are solely those of the authors and do not necessarily represent those of their affiliated organizations, or those of the publisher, the editors and the reviewers. Any product that may be evaluated in this article, or claim that may be made by its manufacturer, is not guaranteed or endorsed by the publisher.

## Footnotes

^1^Centers for Disease Control and Prevention, C. *La Crosse Encephalitis: Epidemiology and Geographic Distribution*. Available online at: https://www.cdc.gov/lac/tech/epi.html (accessed February 12, 2022).
